# Prevalence of Congenital Anomalies in an Indian Maternal Cohort: Healthcare, Prevention, and Surveillance Implications

**DOI:** 10.1371/journal.pone.0166408

**Published:** 2016-11-10

**Authors:** Prajkta Bhide, Pooja Gund, Anita Kar

**Affiliations:** School of Health Sciences, Savitribai Phule Pune University, Pune, 411 007, India; University of New South Wales, AUSTRALIA

## Abstract

**Background:**

India lacks a national birth defects surveillance. Data on the prevalence of congenital anomalies are available mostly from hospital-based, cross-sectional studies. This is the first cohort study from India, where 2107 women were followed till pregnancy outcome, in order to measure the prevalence and types of congenital anomalies, their contribution to neonatal mortality, implications for surveillance, and the health service needs for prevention and management.

**Methods:**

The study followed a cohort of 2107 pregnant women till outcome which was miscarriage, termination of pregnancy, live or stillbirth, neonatal and post-neonatal mortality. Case ascertainment of congenital anomalies was done through visual examination, followed by various investigations. Rates of congenital anomaly affected births were reported per 10 000 births. Health service needs were described through retrospective analysis of events surrounding the diagnosis of a congenital anomaly.

**Results:**

Among 1822 births, the total prevalence of major congenital anomalies was 230.51 (170.99–310.11) per 10 000 births. Congenital heart defects were the most commonly reported anomalies in the cohort with a prevalence of 65.86 (37.72–114.77) per 10 000 births. Although neural tube defects were two and a half times less as compared to congenital heart defects, they were nevertheless significant at a prevalence of 27.44 (11.73–64.08) per 10 000 births. In this cohort, congenital anomalies were the second largest cause of neonatal deaths. The congenital anomaly prenatal diagnosis prevalence was 10.98 per 1000 births and the congenital anomaly termination of pregnancy rate was 4.39 per 1000 births.

**Conclusions:**

This first cohort study from India establishes that the congenital anomaly rates were high, affecting one in forty four births in the cohort. The prevalence of congenital anomalies was identical to the stillbirth prevalence in the cohort, highlighting their public health importance. The results of this study identify the need for a well defined national programme with components of prevention, care and surveillance.

## Introduction

The Global Burden of Disease study 2013 identified congenital anomalies among the top ten causes of mortality in children less than five years of age [1]. While congenital anomalies are the leading cause of death in children in this age group in the high-income countries, they are not considered to be significant public health problems in low- and middle-income countries (LMICs) [2]. The key reasons for this public health under-prioritization in LMICs relate to the inherent characteristics of these conditions. They are low in prevalence, their proportionate contribution to mortality is significantly lower as compared to other perinatal causes, infections and malnutrition, and their management is resource intensive [3–5]. Due to these characteristics, few LMICs have public health services for birth defects, including congenital anomalies. This public health under-prioritization in LMICs however needs to be revisited for several reasons. Globally, child mortality trends are showing a decrease, with reduction in mortality due to infections and malnutrition [6]. This reduction is likely to be accompanied by a transition in the causes of child mortality in LMICs, with a proportionate increase in non-communicable conditions like congenital anomalies. In LMICs, epidemiological transition is witnessed in the differences in the urban and rural mortality causes and rates. In India for example, urban infant mortality rate (IMR) is 27 as compared to rural IMR of 44 per 1000 live births [7], with sepsis, pneumonia and diarrhoea being significantly larger causes of mortality in rural areas. The transition in causes of mortality in urban areas is likely to be accelerated by the availability of standard maternal and pediatric services. Such services would lead to appropriate management of intrapartum related complications, premature births, low birth weight babies, and infections, resulting in increasing contribution of congenital anomalies to neonatal deaths. The possibility of transition in causes of mortality in urban areas of LMICs argues for the need for data on the magnitude and types of congenital anomalies, and the proportionate mortality due to congenital anomalies in LMICs. Another reason to investigate congenital anomalies in LMICs is that not all congenital anomalies are lethal. Babies born with several types of non-fatal anomalies would survive with disability or need lifelong care, often leading to out-of-pocket and catastrophic expenditure for affected families [4, 5, 8]. There is scant data on the number of live born children with birth defects. Data on the healthcare needs of babies affected with congenital anomalies remains unavailable [4]. Furthermore, as most LMICs have both public and private health services, with significant maternal mobility for antenatal care, data is needed to identify the appropriate methods of establishing surveillance for congenital anomalies.

Like other LMICs, congenital anomalies are not considered to be a priority health problem in India [9]. In 2010, congenital anomalies were estimated to be the fifth largest cause of neonatal deaths in India after preterm births (34.7%), intrapartum complications (19.6%), pneumonia (16.3%) and neonatal sepsis (15%) [6]. Despite this ranking, in absolute numbers, congenital anomalies were estimated to contribute to 60 699 neonatal deaths in India in 2013, which accounted for the highest global burden of neonatal mortality due to congenital anomalies [10]. India lacks a national birth defects surveillance, indicating that there is no data on the magnitude of congenital anomalies in the country. Thus, systematic data on the magnitude of congenital anomalies, the most prevalent types of congenital anomalies, their healthcare impact and their impact on neonatal health are required, especially as India has announced a programme for the management of children born with selected birth defects [11]. It is noteworthy that several studies have attempted to measure the magnitude of congenital anomalies in India [12–18]. Most suffer from methodological issues such as non-random sampling, lack of use of standard case definitions and lack of systematic evaluation of infants. Thus these studies yield a wide variation in prevalence.

In this study, we addressed some of these knowledge gaps, and methodological issues through a cohort study design, which collected data on all congenital anomaly affected pregnancies, including those that were terminated. We measured the magnitude and types of congenital anomalies in a cohort of 2107 urban women attending antenatal clinics at government hospitals in Pune, the eighth largest city in India. We report here the birth prevalence of major congenital anomalies, the birth prevalence by type of anomaly and the contribution of congenital anomalies to public health indicators such as neonatal and perinatal mortality rates. In order to strengthen the completeness of the data, we followed up the birth cohort to identify additional cases that may have been diagnosed post-discharge. Through retrospective analysis, we provide data on the healthcare, surveillance and preventive implications of congenital anomalies.

## Materials and Methods

### Study design and population

A prospective cohort study (the Pune Urban Birth Outcome study, PUBOS) was undertaken wherein 2107 women reporting for first antenatal checkup (ANC) at four government hospitals in Pune city, India, were enrolled and followed till outcome. Women were eligible for enrollment if they presented for registration at less than 16 weeks of gestation, were local residents, provided a telephone number for contact during the follow-up period, and were conversant in the local language (Marathi) or Hindi. Women were recruited into the study from May 2013 to March 2015 and followed up till December 2015 for outcome data collection.

### Ethics

The study was initiated after approval of the Institutional Ethics Committee of Savitribai Phule Pune University. Women were explained the study and invited to participate. While consenting to participate in the study, women were requested to provide permission to access medical records, to be contacted by telephone at least twice during the pregnancy period, and after a minimum period of one month post delivery. Written informed consent was obtained from the women prior to their enrollment in the study. Illiterate women were asked to provide left-hand thumb impression in lieu of signature, in the presence of a witness as per standard guidelines [19]. The data was coded to maintain confidentiality during analysis. Relevant governance permissions were obtained prior to initiating the study.

### Data collection

Data on pregnancy outcomes were monitored during the follow-up period through telephone contacts and verified from delivery room records, discharge cards and pregnancy termination records. In order to ensure that all anomalies were recorded, the doctors and nursing staff at the obstetric/gynecology department were informed of the study, and posters with images of common anomalies were put up in the nurses’ room in the delivery wards. A member of the research team was stationed at each of the hospitals during the study period. There was 9% loss to follow up at the end of the study period. For estimation of neonatal mortality, the birth cohort was further followed up in the post-neonatal period and data was collected on the viability of the baby and cause of death, if known, in case of demise. There was a 13% loss to follow up in this period. Out of the 1536 babies that were followed up, 78% were contacted three months after birth. The caregiver was asked about the health of the baby in order to identify any anomalies that may have been diagnosed after discharge post delivery.

### Categorization of congenital anomalies

Congenital anomalies were diagnosed through physical examination, or through various investigations (ultrasonography, 2D echocardiography). Anomaly data was abstracted from prenatal ultrasound reports, neonatal case sheets and other clinical records. Anomalies were assigned International Statistical Classification of Diseases and Related Health Problems (ICD-10) codes by the researchers in order to facilitate system-wise classification of anomalies [20]. The overall categorization of anomalies for inclusion in the analysis was done using the European Surveillance of Congenital Anomalies (EUROCAT) guidelines [21]. These guidelines were also used to categorize anomalies as major or minor.

### Calculation of rates

Coded data was entered into Statistical Package for Social Sciences version 17.0 (SPSS 17.0) and analysed. Stillbirth was defined as a baby born with no signs of life at or after 22 weeks of gestation. Stillbirth rate was reported as the number of stillbirths per 1000 births. Neonatal mortality rate was reported as the number of neonates dying before reaching 28 days of age, per 1000 live births. Perinatal mortality rate was estimated as the number of stillbirths and deaths in the first week of life (early neonatal deaths) per 1000 births. Prevalence of congenital anomalies was calculated as per the World Health Organization birth defects surveillance manual [22]. Total prevalence of congenital anomalies (per 10 000 births) was calculated as the total number of cases of congenital anomalies including live births, stillbirths and elective terminations of pregnancy for fetal anomaly (ETOPFA) divided by the total number of births (including both live and stillbirths). Live birth prevalence (per 10 000 live births) was calculated as the number of live births with congenital anomalies divided by all live births. A fetus/baby with multiple anomalies was counted once within each class of anomaly. Other public health indicators were reported as per EUROCAT [23]. The congenital anomaly prenatal diagnosis prevalence (per 1000 births) was calculated as the number of prenatally diagnosed cases divided by the total number of births (both live and stillbirths). Congenital anomaly termination of pregnancy rate was reported as the number of elective terminations of pregnancy for fetal anomalies per 1000 births. In addition, prevalence per 1000 births was also calculated for selected anomalies typically requiring surgery as identified in the EUROCAT data [23].

## Results

### Pregnancy outcomes

Of the 2107 women recruited in the study, data on pregnancy outcomes were available for 1910 women. The characteristics of the pregnancy cohort have been described previously [24]. There was a 9% loss to follow up as these women did not access services at the registered hospital, and could not be further contacted. The outcome data is presented in Table 1. There were a total of 1929 outcomes among these 1910 women (including 19 sets of twin pregnancies), of which 1781 (92.4%) were live born and 41 (2.1%) were stillbirths (stillbirth rate 22.5 per 1000 births). Among singleton live births, 20% (342/1701) were low birth weight babies, while there were 11% (183/1734) preterm births. Sixty-one percent (1100/1803) of the women delivered at the hospital where they had registered for ANC, and the remaining women delivered at other hospitals. There were 25 unintended home births/births during transportation to facility. There were 22 neonatal deaths (neonatal mortality rate 12.35 per 1000 live births) majority of which occurred in the early neonatal period. The perinatal mortality rate was 34 per 1000 births. There were 107 pregnancy terminations (98 miscarriages including spontaneous abortions, missed abortions and threatened abortions, in addition to eight ETOPFA). In terms of total outcomes occurring in this cohort, one in five births were low birth weight, one in nine were preterm births, one in 20 pregnancies resulted in a miscarriage and stillbirths affected one in 44 births.

**Table 1:** Pregnancy outcomes in the cohort (n = 2107).

Outcomes	N	Rates	Outcomes with major anomalies	Congenital anomaly affected rates
Total outcomes	1929		42	230.51 per 10 000 births
Live births	1781 (92.4%)		30	168.44 per 10 000 live births
Neonatal deaths	22	12.35 per 1000 live births	7 (31.8%)	3.93 per 1000 live births^a^
Early neonatal deaths	21	11.81 per 1000 live births	6 (28.6%)	3.36 per 1000 live births
Late neonatal deaths	1	0.6 per 1000 live births	1 (100%)	0.56 per 1000 live births
Stillbirths	41 (2.1%)	22.5 per 1000 births	4 (9.75%)	2.19 per 1000 births^b^
Perinatal deaths	62	34 per 1000 births	10 (16.13%)	5.5 per 1000 births
Terminations of pregnancy (inc. spontaneous abortions, missed abortions, threatened abortions and after detection of fetal anomalies^c^)	107 (5.5%)		8 (7.48%)	4.39 per 1000 births

^a^ includes one case each of tracheoesophageal fistula, diaphragmatic hernia, two cases of polycystic kidney disease, one case of multiple malformations (genital and musculoskeletal system involvement) and two cases with incomplete anomaly information

^b^ includes one case each of duodenal atresia, spina bifida, congenital cystadenomatoid malformation of the lung and one case of multiple malformations (nervous, circulatory and musculoskeletal system involvement)

^c^ includes one case each of anencephaly, spina bifida, congenital cystadenomatoid malformation of the lung, hyperechogenic bowel, bilateral renal agenesis, bladder outflow obstruction, one case of multiple malformations (nervous and musculoskeletal system involvement) and one fetus with incomplete anomaly information

### Prevalence of major congenital anomalies

There were 42 major anomaly affected cases (total prevalence of congenital anomalies 230.51 (170.99–310.11) per 10 000 births). Among these, 30 were live births (rate 168.44 (118.24–239.44) per 10 000 live births) and 4 were stillbirths (rate 2.19 (8.54–56.31) per 1000 births). Among congenital anomaly affected live births, prematurity was 17% (5/30), and 38% (10/26) of these babies had low birth weight.

### System-wise prevalence of major congenital anomalies

Table 2 indicates the system-wise prevalence of major congenital anomalies. The data showed that congenital heart defects (CHDs) were the most prevalent anomalies (65.86 per 10 000 births), with atrial septal defects (43.91 per 10 000 births) and ventricular septal defects (27.44 per 10 000 births) being the most commonly presenting heart defects. Two-thirds of the CHDs were detected post birth at mean age 4.5 ± 2 days. Malformations of the musculoskeletal system (49.40 per 10 000 births) were primarily contributed by talipes equinovarus (32.93 per 10 000 births). Urinary system anomalies (38.42 per 10 000 births) included congenital hydronephrosis (16.47 per 10 000 births) and polycystic kidney disease (10.98 per 10 000 births). The most frequent nervous system anomalies were neural tube defects (NTDs, 27.44 per 10 000 births). Anomalies of the digestive system, genital organs and respiratory system were less frequently encountered (21.95 per 10 000 births, 16.47 per 10 000 births and 10.98 per 10 000 births respectively). In terms of all births, the data implied that one in 44 births was affected with a major congenital anomaly, one in 152 births was affected with a congenital heart defect, one in 304 births was affected with talipes or a renal anomaly, while one in 364 births presented with a neural tube defect.

**Table 2:** System-wise classification of anomalies.

Affected system	Anomaly^a^	Number of cases	Prevalence per 10,000 births	Number of affected live births	Prevalence per 10 000 live births
All anomalies		42	230.51 (170.99–310.11)	30	168.44 (118.24–239.44)
Congenital heart defects		12	65.86 (37.72–114.77)	11	61.76 (34.52–110.26)
	Complex congenital heart defects	2	10.98 (3.01–39.94)	2	11.28 (3.08–40.85)
	Atrial septal defect	8	43.91 (22.27–86.40)	8	45.12 (22.78–88.39)
	Ventricular septal defect	5	27.44 (11.73–64.08)	5	28.2 (12.00–65.55)
	Other (includes one case each of double outlet right ventricle, single ventricle, tricuspid atresia, pulmonary valve atresia, aortic valve atresia and two cases of atrioventricular septal defect)	7	38.42 (18.62–79.09)	6	33.69 (15.45–73.31)
Musculoskeletal system anomalies		9	49.4 (26.01–93.61)	7	39.3 (19.05–80.91)
	Talipes equinovarus	6	32.93 (15.10–71.66)	4	22.46 (8.74–57.61)
	Other (includes one case each of diaphragmatic hernia, cranio-facial dysmorphism, congenital scoliosis)	3	16.47 (5.60–48.30)	3	16.47 (5.73–49.41)
Urinary system anomalies		7	38.42 (18.62–79.09)	5	28.2 (12.00–65.55)
	Congenital hydronephrosis (>10 mm)	3	16.47 (5.60–48.30)	3	16.92 (5.73–49.41)
	Polycystic kidney disease	2	10.98 (3.01–39.94)	2	11.28 (3.08–40.85)
	Other (includes one case each of bilateral renal agenesis and bladder outflow obstruction)	2	10.98 (3.01–39.94)	-	-
Nervous system anomalies		5	27.44 (11.73–64.08)	-	-
	Neural tube defects including anencephaly (1), spina bifida (3) and encephalocele (1)	5	27.44 (11.73–64.08)	-	-
	Other (includes one case of holoprosencephaly and two cases of Dandy Walker malformation)	3	16.47 (5.60–48.30)	-	-
Digestive system anomalies	includes one case each of tracheoesophageal fistula, duodenal atresia, hyperechogenic bowel and cleft palate	4	21.95 (8.54–56.31)	2	11.28 (3.08–40.85)
Genital anomalies		3	16.47 (5.60–48.30)	3	16.92 (5.73–49.41)
	Hypospadias	2	10.98 (3.01–39.94)	2	11.28 (3.08–40.85)
	Indeterminate sex	1	5.49 (0.97–31.02)	1	5.64 (0.99–31.74)
Respiratory system anomalies	Congenital cystadenomatoid malformation of the lung	2	10.98 (3.01–39.94)	-	-
Incomplete information to classify		4	21.95 (8.54–56.31)	3	16.92 (5.73–49.41)

^a^ Multiple anomalies have been counted once in each class

### Contribution to neonatal and perinatal mortality

Of the 22 neonatal deaths, 41% (9/22) were due to prematurity, 32% (7/22) due to congenital anomalies, 9% (2/22) due to birth asphyxia and one was a case of milk inhalation. The causes of three neonatal deaths could not be identified as discharge records were unavailable with the family. The neonatal mortality rate due to major congenital anomalies in the cohort was 3.93 per 1000 live births, that is, they contributed to one-third of neonatal deaths. The early neonatal mortality rate due to major congenital anomalies was 3.36 per 1000 live births, with the deaths being attributed to two cases of renal anomalies, and one case each of diaphragmatic hernia and tracheoesophageal fistula. The perinatal mortality rate due to major congenital anomalies was 5.5 per 1000 births, that is congenital anomalies contributed to one-sixth (10/62) of the total perinatal mortality. Congenital anomalies were responsible for 7.5% (8/107) of the pregnancy terminations.

### Minor anomalies

In addition, there were 32 minor anomaly affected live births, of which the most frequently presenting anomalies were unilateral undescended testicle and single umbilical artery (prevalence 28.07 per 10 000 live births each). Other common minor anomalies included natal teeth and ear anomalies (16.84 per 10 000 live births each).

### Other birth defects

The birth cohort was followed up and caregivers of 1536 infants were re-contacted after three months post delivery and probed for post-discharge diagnosis of any congenital anomaly. The follow up revealed no further congenital anomaly affected infants. However, there was one case each of Down syndrome, congenital hypothyroidism, thalassaemia and one post-neonatal demise after a diagnosis of Leigh syndrome.

### Health service implications

The anomalies identified as typically requiring surgery included severe CHDs, orofacial cleft, diaphragmatic hernia, duodenal atresia and tracheoesophageal fistula. In the event that surgery was offered for all these infants, the prevalence of congenital anomalies typically requiring surgery would have been 3.29 per 1000 births. Out of 11 live births with CHDs, two cases were complex defects and the parents were informed of the severity of the defect. Both babies expired in the post-neonatal period. Among the remaining nine babies with CHDs, three resolved by three to four months of age, while the remaining six required surgery. Thus in this cohort, the cardiac surgery rate would have been 3.29 per 1000 births. Other conditions which may require medical interventions in infancy or early childhood included talipes equinovarus (22.46 per 10 000 live births), congenital hydronephrosis (16.84 per 10 000 live births) and hypospadias (11.23 per 10 000 live births). Among the minor anomalies, conditions likely to require referral to pediatric services included undescended testicle (28.07 per 10 000 live births) and ear anomalies (16.84 per 10 000 live births).

### Contribution to childhood disability and chronic medical conditions

There were 23 out of 42 (55%) infants with major congenital anomalies who survived beyond the neonatal period. There were three deaths in the post-neonatal period as a consequence of the anomaly and three cases of CHD resolved spontaneously. The 17 surviving infants with major congenital anomalies included six infants with CHDs, four infants with talipes equinovarus, three infants with congenital hydronephrosis, two with hypospadias and one each with orofacial cleft and scoliosis. In addition, there was one infant each with a diagnosis of Down syndrome, thalassaemia and congenital hypothyroidism.

### Prenatal detection and counseling service implications

The congenital anomaly prenatal diagnosis prevalence for major anomalies was 10.98 per 1000 births. Among the 42 women with anomaly affected pregnancies, complete ultrasonography usage data was available for 35 women. Among these, 19 (54%) had been detected with an anomaly affected pregnancy during the gestation period (one in the first trimester, 14 in the second trimester and four in the third trimester). Of these, eight pregnancies were terminated, four resulted in stillbirths and seven were live births. Congenital anomaly ETOPFA prevalence rate was 4.39 per 1000 births with NTDs and renal anomalies contributing to half of all the terminations due to congenital anomalies. The average time period between diagnosis of the anomaly and pregnancy outcome was 12 ± 2.5 weeks among live births, 4.5 ± 1 weeks for stillbirths and 3.5 ± 1 days for terminations of pregnancy for fetal anomaly.

### Surveillance implications

Among 30 live births with major anomalies, majority (25/30, 83%) were delivered at the place of registration, or were referred to a higher institution for delivery due to pregnancy complications (cervical cerclage, oligohydramnios and after prenatal detection of anomalies). Five (17%) women delivered at hospitals other than the place of registration. Overall, 34 out of the 42 (81%) major anomaly affected outcomes occurred at the place of registration. The limitations of hospital based, passive surveillance was reflected as follows. As 39% women had delivered elsewhere (hospitals other than the place of registration, at home, or in-transit), the anomaly rate calculated at the place of registration was 305.75 per 10 000 births (34/1112). This is an over-estimate as compared to the rate of 230.51 per 10 000 births (42/1822) obtained through the cohort study.

## Discussion

### Main findings of this study

This study was initiated as there is no national surveillance to measure the magnitude of congenital anomalies occurring in India, and previously conducted cross-sectional studies have yielded wide differences in overall prevalence rates. In this background, this study adopted a cohort study design to measure the birth prevalence of congenital anomalies. The study further followed up the birth cohort in order to determine if there were additional cases diagnosed after discharge.

### Generalizability of the data

The rates obtained in this cohort were first compared to available local and national level indicators in order to test the generalizability of the data obtained. The neonatal mortality rate (12.35 per 1000 live births) in the cohort was similar to the data of the population from which the sample of women had been drawn (Maharashtra state, urban neonatal mortality rate 11 per 1000 live births and national urban neonatal mortality rate 15 per 1000 live births) [7]. The stillbirth rate (22.5 per 1000 births) was also similar to the estimated national stillbirth rate of 23 per 1000 births [25], and to that of Maharashtra state, that is 2.2% [26]. This indicated that the data from this study could be generalized to the larger population, keeping in mind that congenital anomaly rates would vary with exposure rates.

### Magnitude of anomaly affected pregnancies

The total birth prevalence of congenital anomalies in this cohort was 230.51 (170.99–310.11) per 10 000 births, as compared to 215.54 (214.14–216.94) per 10 000 births from the EUROCAT [27]. Assuming that the rate of 2.3%to hold true for the country, in absolute numbers, congenital anomalies would affect 589 990 (437 674–793 445) births in the country each year (total number of births 25 595 000) [28]. Thus, the first major finding of the study was the significant congenital anomaly rate, implying that congenital anomalies are not insignificant in terms of the number of affected births. The prevalence by type of anomaly showed known global trends, with congenital heart defects being the most prevalent type of birth defect. It is notable that at birth, the prevalence of congenital heart defects was 61.76 per 10 000 live births, that is 1.5 fold lower than the estimated prevalence of 9.3 per 1000 live births for Asia [29]. This observation implies that the majority of diagnosis must be occurring at later ages. Congenital heart defects affected one in 152 births, suggesting that there may be as many as 168 569 children born with CHD in India each year. As compared to CHDs, the absolute numbers of NTDs would be lower, but not insignificant as at a birth prevalence of 27.44 per 10 000 births, these conditions would affect 70 233 births in the country annually.

### Health service implications

The data from this study has significant health service implications in terms of necessary care, and referral needs. In this cohort, several babies with anomalies needed care, in the form of corrective surgery, orthopedic interventions or additional investigations like imaging. These included newborns with CHDs, talipes equinovarus, orofacial cleft, hypospadias, congenital hydronephrosis, undescended testicles, diaphragmatic hernia and tracheoesophageal fistula. Keeping in mind absolute numbers as indicated in this study, the data suggests that pediatric surgery services for congenital anomalies have to be included as a component of newborn care. At present, corrective surgery for CHDs is available free of cost only to families below the poverty line [11]. For other surgeries, and for families above the poverty line, these conditions would require out of pocket expenditure.

The second health care implication was in the utilization of sonography services for the detection of fetal anomalies. In this study, eight out of 42, that is nearly one fifth of severely affected births were avoided through prenatal detection and subsequent pregnancy termination. Although termination of pregnancy due to detection of a fetal anomaly is legally permissible in India, there are currently no government guidelines on the incorporation of sonography as a routine investigation in antenatal care. Furthermore, a need for counseling prior to and after the ultrasound examination was observed, as women underwent this investigation with little preparation in case of detection of an anomaly. It was noted that upon detection of an anomaly, and when there was no option to terminate the pregnancy, women were left to carry the pregnancy to term, with no psychosocial support to help them address or prepare for the impending birth. Thus, the study observed the need for guidelines for the use of sonography during pregnancy, including appropriate pre- and post-test counseling, accompanied by widespread dissemination of knowledge among women about the utility of sonography in the detection of congenital anomalies.

The other required health service intervention was the need for a linkage between pediatric services and care for children with special needs. There are currently no rehabilitative services for children with disabilities, and as such there is no opportunity for referral of children with special needs for rehabilitative care. In the same lines, it was observed that a psychosocial support service for parents was urgently required. In lieu of available services, several parents turned to the research team for knowledge and advice.

### Surveillance

The current study identified two important lessons for birth defects surveillance in India. These were the limitations of using hospital based registry data for estimation of prevalence rates, and the need to implement standard case definitions. In the present study, almost 20% major anomaly affected outcomes had occurred at places other than the place of registration. Without follow up of these women, the anomaly rate would be calculated at 305.75 per 10 000 births as against 230.51 per 10 000 births. This observation suggests that hospital based registries may provide best-available data, but may result in over- or under-estimation of anomaly affected births, due to lack of defined denominators. The difference in the prevalence of NTDs observed in this cohort (27.44 per 10 000 births) and that obtained in an earlier systematic review and meta-analysis of hospital-based studies (4.1 per 1000 births) [30] reinforces this point. The second learning was the need to implement stringent case definitions and implement surveillance for selected anomalies. Most Indian studies report the highest prevalence of nervous system anomalies and anomalies of the musculoskeletal system [12–18]. This is in contrast to the data from this cohort study, where congenital heart defects along with musculoskeletal system anomalies were most prevalent. This underlines the need for ensuring a strict quality of surveillance data, as incorrect and incomplete recording of cases can misinform health policies and priorities.

### Impact on public health indicators

EUROCAT has identified a set of six indicators which can be used to assess the impact of congenital anomalies on public health indicators [23]. These include perinatal mortality rate, congenital anomaly prenatal diagnosis prevalence, congenital anomaly ETOPFA prevalence, and the birth prevalence of anomalies typically requiring surgery. While these are important health service indicators, perhaps the most significant public health indicator from the perspective of LMICs would be the neonatal mortality rate due to congenital anomalies. This indicator could be used to monitor the transition in causes of mortality, and anticipate the type and quantity of birth defects services that would be needed. In this context, it is important to realize that congenital anomalies may be contributing to two key public health indicators of LMICs, low birth weight and prematurity. In this cohort, 17% of congenital anomaly affected babies were preterm, while 38% had low birth weight. With specific reference to India, the observation that congenital anomalies were the second largest cause of neonatal mortality in this study also indicates the need to study causes of infant mortality in other urban areas of India. Such studies are needed to monitor whether transition in causes of neonatal mortality has started occurring across different geographic, socio-economic and healthcare settings of the country.

### Implications for a birth defect policy

This study has several implications for a national policy to address congenital anomalies and other birth defects, not only in India, but also in other similar developing countries. The key findings from the study suggest that the first requirement for a birth defects policy is the need to establish surveillance for birth defects. Surveillance data will permit the description of the epidemiology and public health impact of congenital anomalies, and anticipate the health care needs for birth defects. With epidemiological transition underway, surveillance should provide data disaggregated for urban and rural births, as the risk exposures are likely to be different in both these settings. Surveillance should also provide data on the number of infants with special needs, in order to provide support for childhood disability and children with chronic medical needs. In this context, child health programmes have to expand from their traditional focus, to address the rights of children with special needs. Underlining the surveillance, is the need to thoroughly examine infants prior to discharge, and implementation of low cost measures such as pulse oximetry to ensure that most babies with CHD are detected at the earliest [31]. A best available package of services for birth defects needs to be established. An integral part of the service package would be the provision of counseling and psychosocial support, in order to address the distress associated with an affected birth, and to limit out-of-pocket expenditure on multiple medical advices. The Rashtriya Bal Swasthya Karyakram, the national early screening and diagnosis programme for children in India, has in place some services to address a few of these concerns [11].

As important as surveillance is the prevention of congenital anomalies. A recent study has reported the high prevalence of periconception risk factors for adverse pregnancy outcomes including birth defects among urban Indian women, especially those who were poorly educated and from low socioeconomic backgrounds [24]. In such a scenario, preconception interventions take on a very important role in the prevention of birth defects. For example, assuming that upto 40% of NTDs can be prevented with preconception folic acid supplementation [32], the intervention would reduce about 30 000 affected births in India, considering complete compliance. The cost-benefit implications of folic acid supplementation in India however need to be carefully considered. Keeping in mind that a significant number of women from developing countries are likely to have low education levels, widespread health promotion messages emphasizing preconception care to prevent birth defects should be a key step in any birth defects prevention programme. As however, the main focus of maternal health services in developing countries is on the provision of antenatal care, programmatic changes are required so that women can be reached prior to conception.

The strengths of our study were in the cohort design, the inclusion of data on pregnancy terminations due to congenital anomalies, and follow up of the birth cohort in order to determine additional anomaly diagnoses after the neonatal period. Our study also had a few limitations. Firstly, we had a 9% loss to follow up in the measurement of pregnancy outcome. There was a further 13% loss to follow up post delivery in the birth cohort. Although our sample size was small, the prospective design provides the first data on the magnitude of congenital anomalies in the country. Even though we compared key national and state-level indicators with those obtained in this cohort, the generalizability of the data needs to be carefully considered in the background of the variation in risk exposures that is likely to exist across the country.

## Conclusions

The findings of this study indicate that congenital anomalies are not insignificant conditions, as their birth prevalence rate is equivalent to global rates. Extrapolating our findings for India, our data suggest 589 990 (437 674–793 445) affected births, 168 569 (97 261–294 342) cases of CHDs and 70 233 (30 714–163 808) cases of NTDs each year in the country. The findings of this study suggest that in urban areas, a transition in the causes of neonatal mortality may be occurring, with increasing contribution of congenital anomalies to neonatal deaths. The data identifies that due to the relative high frequencies of congenital anomalies, birth defects services including components of care, prevention and surveillance in the form a well defined programme are needed in the country.
